# Value, Market Preferences and Trade of Beche-De-Mer from Pacific Island Sea Cucumbers

**DOI:** 10.1371/journal.pone.0095075

**Published:** 2014-04-15

**Authors:** Steven W. Purcell

**Affiliations:** National Marine Science Centre, Southern Cross University, Coffs Harbour, New South Wales, Australia; Leibniz Center for Tropical Marine Ecology, Germany

## Abstract

Market preferences of natural resources contribute to shape their exploitation and production. Beche-de-mer, the product after gutting, cooking, salting and drying sea cucumbers, is exported worldwide to Asian dried seafood markets. A better understanding of the trade, value and market preferences of Pacific island beche-de-mer could identify critical postharvest processing techniques and management strategies for fisheries and aquaculture. Data were collected on export prices and trade of beche-de-mer from Kiribati, Fiji, Tonga and New Caledonia, and the selling prices, respective sizes and organoleptic properties of the products in stores in China. Export prices varied considerably within and among the four countries and low-value species were the most exported by volume. Most of the beche-de-mer from the four Pacific islands is exported to Hong Kong, where quality products are sold and others are distributed to mainland China. Prices of the beche-de-mer in Chinese stores varied up to ten-fold and were mostly influenced by species, body size and, to a lesser extent, physical damage to the products. Market prices across species (averaging US$15–385 kg^−1^) appear to have mostly increased six- to twelve-fold over the past decade. The data allude that fisheries for *Holothuria scabra*, *H. lessoni*, *H. fuscogilva*, *H. whitmaei* and *Thelenota ananas* should be most carefully managed because they were the highest-value species and under greatest demand. The relationships between size of beche-de-mer and sale price were species specific and highly varied. This study also highlights the need for better regulations and/or enforcement of minimum size limits in sea cucumber fisheries, which can help to maximise economic benefits of wild stocks.

## Introduction

### Exploitation and Trade of Sea Cucumbers

The market for luxury seafood commodities in Asia, such as shark fin and sea cucumbers, strongly affects exploitation of wild stocks at a global scale [1], [2]. With fisheries worldwide expanding to lower trophic-order species [3] and the market pressures of mounting affluence [2] and seafood consumption in China [4], sea cucumbers have attracted much interest in export-oriented fisheries in at least 70 countries [5]. Sea cucumbers have been fished for more than 170 years from Pacific islands [6], where at least 20 species are commonly harvested [7], [8]. Recent exploitation in many Pacific islands has been intense, leading to depletion of stocks and closure of fisheries [7], [9], [10]. The only Pacific island fisheries currently open to export-oriented fishing are Fiji, Tonga, Kiribati, New Caledonia, French Polynesia and Vanuatu (recently re-opened) [9].

Aquaculture of tropical sea cucumbers has been expanding rapidly. Presently, the high-value sandfish *Holothuria scabra* is the tropical species of greatest interest, and aquaculture trials of more than a half dozen other species have proved promising [11]. Profitability is predicated on harvesting the animals at a size to give optimal economic yield relative to grow-out costs [11].

After being collected from reefs and soft-bottom habitats, or harvested from farms, fishers or farmers either process sea cucumbers themselves or sell them to commercial processors/exporters who do the work [12], [13]. Processing generally involves cutting and gutting the animals, then cooking, salting, smoke curing, and drying [6], [13], [14]. Dried sea cucumbers are then exported to Asian market entrepôts, predominantly Singapore, Taiwan and Hong Kong [15], [16]. Important questions for fisheries are to which markets are the products exported, which species are most valuable and what factors most affect market prices? Understanding these aspects could allow fishery management institutions to optimise fishing regulations and training of fishers in post-harvest processing.

### Consumption of Sea Cucumbers

**Figure 1:**
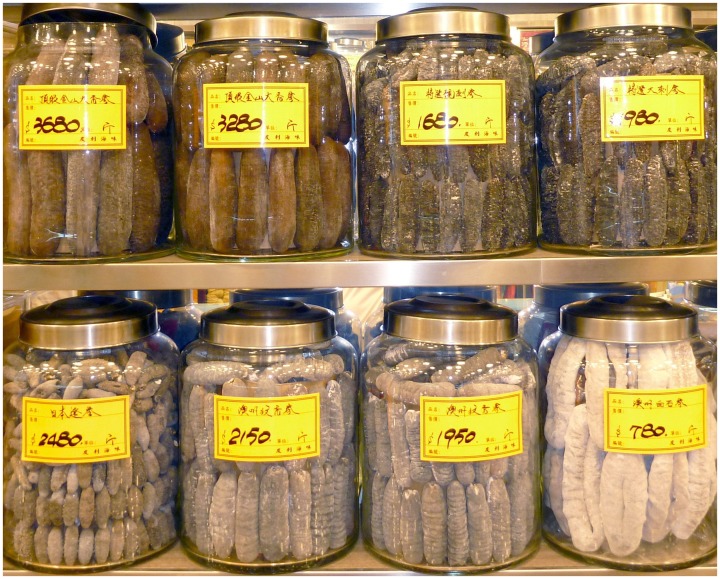
High-value tropical beche-de-mer on sale in jars in Hong Kong. Upper-left: *H. lessoni*; lower-right: *H. fuscogilva*; lower-centre: *H. scabra*. Photo: S.W. Purcell.

Sea cucumbers are consumed predominantly by Chinese and other southeast Asians such as Singaporeans, Vietnamese, Koreans, Malaysians, and Japanese [17], [18]. The dried product, known as beche-de-mer or trepang (Fig. 1), is rehydrated and eaten avidly for its health and medicinal benefits [19], [20]. Traditional Chinese medicine touts that sea cucumbers have healing properties, especially for joint ailments, urinary problems and certain cancers [19], [21]. Recent studies have indeed shown sea cucumbers to be high in protein, important amino acids, and certain bioactive components such as mucopolysaccharides and chondroitin sulphate [19], [22]. Sea cucumbers are also one of the delicacies of fine Chinese cuisine and are of cultural importance [18], [20].

### Market Preferences

Asian consumers are rather discerning about product quality [14],[23],[24]. Hong Kong and Guangzhou (Canton) are geographically close in southeast China and the preferences for seafoods at these entrepôts reflect Southern Chinese cuisine [20], namely Cantonese style. While sea cucumbers are often cut up for sauced dishes, they are sometimes served whole. Hence, the size, colour, shape and appearance of the products are valued by consumers [14], [24]. Different species also have different tastes and textures, and certain species are used for certain dishes. A common notion is that bigger sea cucumbers are worth more and this is often shown in the prices that traders offer to fishers [23], [25], [26]. However, data have not been published to show whether this is true in the marketplace across a range of species.

Prices in the marketplace are likely to vary depending on a wide range of factors [14], [23], [26], which may be influential for some species but not others. Such information is valuable to fishers and processors in producing countries so that they can process the animals in the best ways to yield attractive products for export. Understanding price structure across species is important for resource management because market value strongly influences exploitation pressure and the extinction risk on sea cucumbers [27]. The relationship between body size and market price could also be useful for setting size limits in fisheries and is required for optimising the size at which to harvest farmed sea cucumbers [11].

The goal of the study was to provide information support to fishers, processors/exporters, aquaculturists, resource managers and development agencies. The aims were twofold: (1) to determine the trade destinations and value of sea cucumbers from Pacific islands; (2) to determine the prices in Chinese markets for Pacific species and examine which organoleptic properties of the products had greatest influence on market prices. This study incorporates questionnaire data from exporters in four Pacific island countries (Kiribati, Tonga, New Caledonia and Fiji) and data on beche-de-mer in stores in southern China. The findings are relevant across the Indo-Pacific because most of the study species are also harvested and exported from fisheries in the Indian Ocean and Southeast Asia [8]. The study provides economic rationale for minimum size limit regulations for fisheries and an empirical basis for cost-benefit analyses of optimal size-at-harvest for aquaculture. The analyses support future training of fishers in best-practice methods of processing sea cucumbers for export to Asian markets.

## Materials and Methods

### Exports and Trade from Pacific Islands

Data were collected from commercial sea cucumber exporters in Tonga (2011; *n* = 9), Fiji (2011; *n* = 3), Kiribati (2011; *n* = 6) and New Caledonia (2007; *n* = 3) using structured questionnaire-based interviews. They were selected haphazardly for the interviews. The exporters buy sea cucumbers from fishers that are either fresh, which they process into the dried form, or fully dried beche-de-mer ready for export; so all the exporters were also processors. The exporters were asked about export prices, import destinations of their exports, species most exported by volume, species of first choice for buying, and problems encountered with the quality of sea cucumbers bought from fishers.

### Market Study in China

The interviews with exporters revealed that beche-de-mer from the four countries are most often exported to Hong Kong and some of the products are then directed to markets in mainland China. Therefore, a study was conducted in October 2011 of sea cucumber products on sale in Hong Kong (5 stores) and Guangzhou (Canton) (11 stores). Stores in Guangzhou sold mainly in wholesale volumes, whereas stores in Hong Kong sold in retail volumes. The stores in Hong Kong sold a narrower range of species, so some of the beche-de-mer from certain species of sea cucumber were present in markets in Guangzhou but not Hong Kong. Access to the products in the stores was facilitated by Asian consultants and a local interpreter.

Beche-de-mer species were identified from knowledge of dried forms and identification guides [8], [28]. Data collection focused on species of sea cucumbers fished in Pacific islands. Beche-de-mer were sold in open bags or bins (‘lots’) with prices per unit weight (per *jīn* [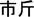 or *shijīn*], equivalent to 500 g, at Guangzhou; per catty [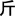 or *kati*], equivalent to 604.79 g, at Hong Kong). Where possible, 2 or 3 lots of beche-de-mer of different grades were sampled haphazardly in each store. Three or four random specimens were selected from each lot. Prices were recorded, then converted using international rates: 1 CNY = 0.159 US$; 1 HKD = 0.129 US$. Specimen lengths were measured to the nearest 0.5 cm along the ventral surface with a ruler and their weights were measured to ±1 g using an electronic balance. For each lot of sea cucumbers, categorical data were recorded to describe three organoleptic (in this case, aspects experienced by sight and smell) properties: the placement of the cut on the specimens, whether they had no smoky smell or a light or strong smoky smell, and whether products were physically damaged. Photographs were taken of some specimens for reference. Discussions were held with ten store owners about product preferences and why certain lots of a species were priced differently to other lots.

The average product (body) lengths of specimens from each lot (bin or bag) in stores were used as the data for analyses. The most appropriate relationships between product length and prices were determined by comparing numerous standard equations using the AIC model selection criterion [29]. Apart from one species, *Holothuria fuscogilva*, the sizes of products found at Hong Kong were mostly larger than those found at Guangzhou because the best products are reserved for the elite Hong Kong market while inferior products are re-exported to mainland China. Analysing data from the two markets separately would yield flawed comparisons because the product sizes were not comparable between locations. The relationships between product length and price appeared valid for both entrepôts, but the potential bias of combining the data is discussed later.

I tested the effects of product factors on the price per kg (*Ln*-transformed) of beche-de-mer in Chinese stores using a General Linear Model (GLM) analysis using SPSS 21, employing a model reduction process. The GLM incorporated continuous (product length) and categorical (species, smoky smell, broken, correct cut) factors. Species with less than three replicates were omitted, leaving eleven species for the analysis. Type III sum of squares was employed, since it is suitable for unbalanced designs with no empty cells. Homogeneity of variances was verified for each model using Levene’s test (*p* = 0.30–0.70). The first model included all main effects and the species*length interaction term, which was subsequently excluded (*F*_10,72_ = 0.64, *p* = 0.78). The main effects of smoky odour was then removed (*F*_2,82_ = 0.59, *p* = 0.56) and cut placement was removed (*F*_1,84_ = 0.74, *p* = 0.39) in the third model. The final model (containing species, specimen length and physical damage) retained ‘physical damage’ as a predictive factor so that the analysis would compare prices for each species on the same proportion of lots that were damaged.

### Ethics Statement

This questionnaires and interviews in this study were conducted under Human Research Ethics approval #ECN-11-040 by the Southern Cross University Human Research Ethics Committee, in accord with Australia’s National Statement on Ethical Conduct in Human Research, 2007.

## Results

### Surveys of Commercial Exporters in Pacific Islands

**Figure 2:**
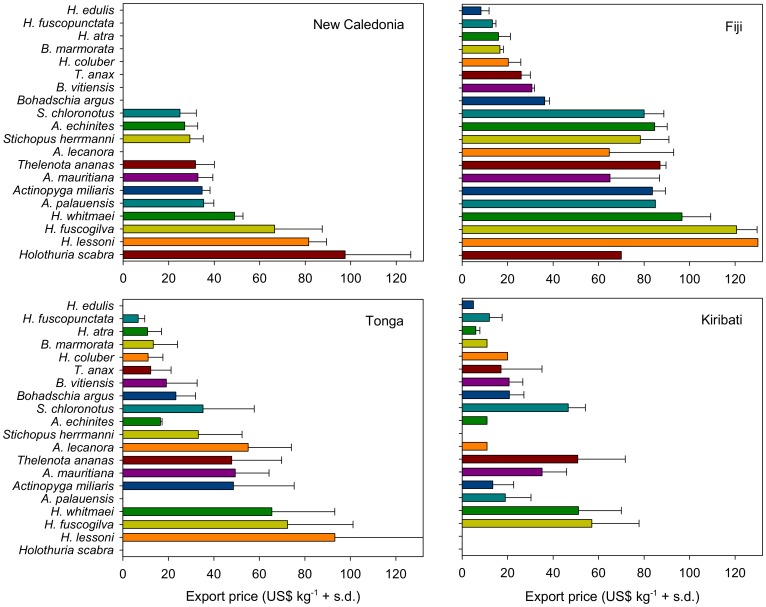
Average export prices of large-sized beche-de-mer from New Caledonia, Fiji, Tonga and Kiribati. Bars without error bars denote singular datum. Missing bars denote that the species was not reported as being exported at the time of the interviews.

The average reported export price of beche-de-mer varied greatly among species and among the four Pacific island countries (Fig. 2). Export prices were, on average, highest in Fiji and lowest in Kiribati. Beche-de-mer from New Caledonia and Tonga yielded comparable prices to exporters but the data from New Caledonia was four years prior to data in the other countries.

**Figure 3:**
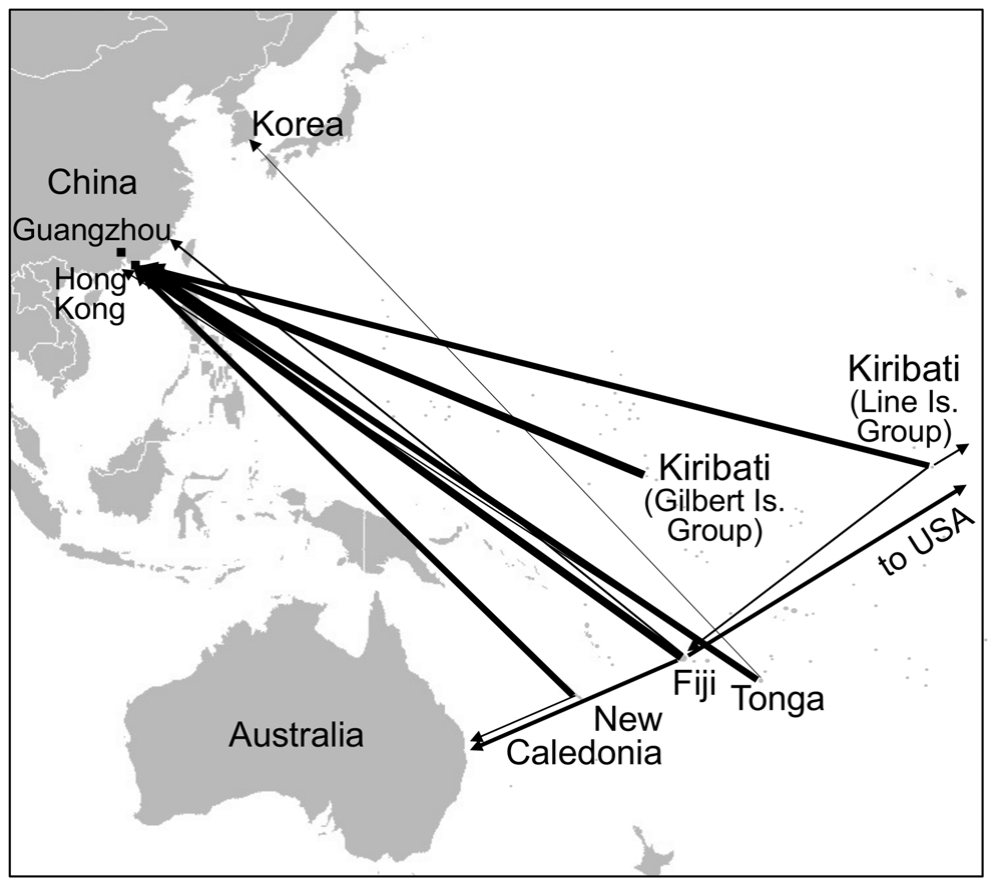
Map of study countries and the destinations of their exports of beche-de-mer. Thicknesses of arrows are scaled to the proportion of exporters exporting product to those destinations.

Hong Kong was by far the most common destination of the exporters from Tonga (82%), Kiribati (75%), Fiji (100%), and New Caledonia (80%) (Fig. 3). Other minor, or less common, destinations were other Chinese cities, Korea, United States and Australia. None of the 21 exporters in the four countries exported beche-de-mer to Singapore or Taiwan.

All three of the Fijian exporters told that amberfish (*Thelenota anax*) was currently the species exported most by volume. Lollyfish (*Holothuria atra*) was most frequently exported most by volume by i-Kiribati exporters. More exporters in Tonga reported surf redfish as being exported most by volume. In New Caledonia, black teatfish (*H. whitmaei*) was most commonly exported most by volume.

In Tonga, Kiribati and Fiji, the species that exporters most commonly (two-thirds) preferred to buy from fishers was white teatfish (*H. fuscogilva*). In New Caledonia, the species most preferred for purchasing from fishers was black teatfish (*H. whitmaei*).

Undersized products from fishers was a regular problem for all exporters in Fiji and Kiribati, for two-thirds in Tonga and one of the three in New Caledonia. Physically damaged sea cucumbers or beche-de-mer from fishers was a regular problem for all exporters in Fiji, 83% in Kiribati, 44% in Tonga and one of three in New Caledonia. Poorly preserved (improperly cooked or salted) beche-de-mer from fishers was a regular problem for two-thirds of exporters in Fiji and Kiribati, one of the three in New Caledonia and none in Tonga.

### Market Study in China

The average store prices of beche-de-mer varied greatly among different lots for a given species and among different species (Table 1). Across species, there was a two-fold to ten-fold variation in prices among products of different lengths. The most expensive product was very large sandfish (*Holothuria scabra*) in one store in Hong Kong, selling for US$1,668 kg^−1^, but I was not permitted to measure the products. Apart from that one lot, golden sandfish (*H. lessoni*) were of similar or higher price, and specimens larger, than sandfish.

**Table 1:** Market value and size descriptors of beche-de-mer species in Chinese stores.

Species	Common name	*n*	Av. Price(US$ kg^−1^) ±s.d.	Max. price(US$ kg^−1^)	Av. Length(cm) ±s.d.	Av. Weight(g) ±s.d.
Hong Kong						
* H. scabra*	Sandfish	12	303^159^	1668	9^2^	41^27^
* H. lessoni*	Golden sandfish	4	385^27^	787	13^1^	104^34^
* H. fuscogilva*	White teatfish	9	192^36^	274	17^2^	214^77^
* H. whitmaei*	Black teatfish	7	180^32^	230	15^3^	232^105^
* S. herrmanni*	Curryfish	2	197^47^	214	15^2^	142^49^
* A. mauritiana*	Surf redfish	1	145	145	10	70
Guangzhou						
* T. ananas*	Prickly redfish	11	130^40^	231	18^3^	194^73^
* H. scabra*	Sandfish	7	137^31^	200	8^1^	28^18^
* H. fuscogilva*	White teatfish	16	120^37^	165	17^5^	264^144^
* S. herrmanni*	Curryfish	5	121^29^	159	11^1^	59^21^
* S. monotuberculatus*	Dragonfish	3	118^13^	133	9^0^	29^2^
* H. whitmaei*	Black teatfish	3	68^43^	116	9^4^	63^42^
* A. palauensis*	Panning’s blackfish	2	106^15^	116	13^3^	149^61^
* S. ocellatus*	Eye-spot curryfish	1	111	111	91	39
* A. lecanora*	Stonefish	3	94^14^	108	10^0^	81^19^
* S. chloronotus*	Greenfish	2	79^22^	95	8^0^	13^3^
* A. spinea*	Burying blackfish	2	79^22^	95	13^0^	92^12^
* A. miliaris*	Hairy blackfish	2	79^22^	95	10^1^	78^13^
* S. horrens*	Dragonfish	2	69^19^	83	7^1^	13^3^
* A. mauritiana*	Surf redfish	5	75^10^	79	10^2^	73^34^
* B. argus*	Leopardfish	3	58^7^	63	18^2^	183^36^
* A. echinites*	Deepwater redfish	1	63	63	10	45
* B. vitiensis*	Brown sandfish	1	48	48	13	107
* H. coluber*	Snakefish	1	38	38	20	115
* T. anax*	Amberfish	5	22^7^	32	20^1^	225^27^
* H. fuscopunctata*	Elephant trunkfish	5	15^3^	19	20^2^	244^64^

Only species fished in Pacific islands are presented. *n* = number of lots (containers). Standard deviations are superscripted.

Across all species, the average market prices for large specimens were, on average, 2.7 times the average export prices (given by island exporters) for similar-sized specimens from the four Pacific islands. The largest proportional increase in average market price relative to export price was for *Holothuria scabra* (4.0), *H. lessoni* (3.8), *Stichopus herrmanni* (3.7) and *Actinopyga lecanora* (3.6). The corresponding increases were smallest for *T. anax* (1.3), *H. fuscopunctata* (1.6) and *A. mauritiana* (1.8).

Compared to Hong Kong, beche-de-mer in Guangzhou were from a more diverse range of species, prices were generally lower, and products of some species were a little smaller (Table 1). Several species known to be fished from Pacific islands were observed in just one store, or not at all, in Hong Kong and Guangzhou.

**Figure 4:**
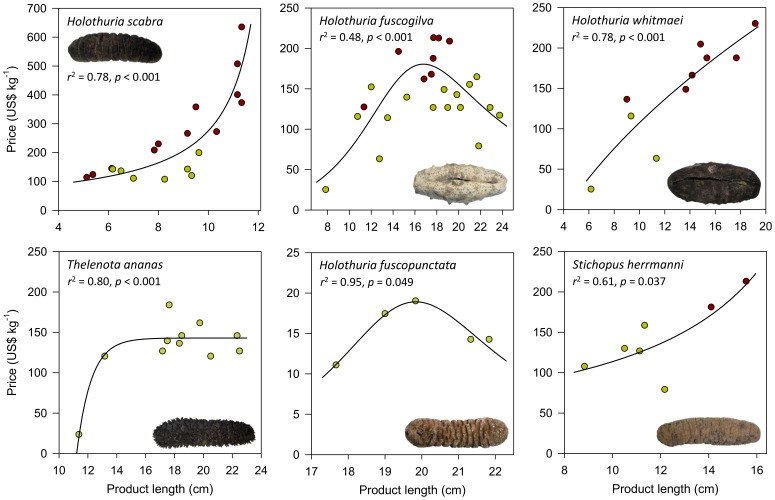
Dried sea cucumber specimen length vs price for six species of beche-de-mer. The scatter plots and curves of best fit by AIC selection are for the species for which there were sufficient data from at least four replicate lots and for which significant relationships were found. All data are average lengths of individual specimens from lots at the same unit price in stores. Dark red dots are data from Hong Kong; green dots are data from Guangzhou.

Price per unit weight tended to increase across a range of specimen lengths for several species: *H. scabra*, *H. whitmaei* and *S. herrmanni* (Fig. 4). For *H. scabra*, there was strong evidence (*p*<0.001) that the effect of specimen length on price was exponential. In contrast, there was strong evidence that price increased to a maximum for 15–19 cm *H. fuscogilva* specimens and relatively weak evidence of a maximum at 19–20.5 cm for specimens of *H. fuscopunctata*, and that prices then decreased with longer specimens for both species. Hence, for those two species, moderately-large specimens seem to attract the best prices. For *Thelenota ananas*, price per unit weight was low for small specimens and relatively consistent at ∼US$150 kg^−1^ for medium-large (17–20 cm) and large (20–23 cm) specimens. There were few data on beche-de-mer of *A. mauritiana* (*n* = 6), *T. anax* (*n* = 5) and *H. lessoni* (*n* = 4), and the relationships between specimen length and price were non-significant.

The final GLM model of effects of product factors on price per kg of beche-de-mer accounted for 79% of the total variation in prices among lots in stores. The analysis reinforced that species and product length had the strongest effects on price (Table 2). The effect of physical damage to the beche-de-mer (e.g. skin broken or burnt) was marginally non-significant, but statistical power (0.53) was low to detect effects. Discussions with store owner/managers indicated that sea cucumbers with smooth and undamaged skin attract higher prices than products with damaged skin. From a statistical viewpoint, a smoky odour to the products and the cut made on for gutting the animals had no discernible effect on prices. However, shop owners told that while a mild smoky smell was acceptable, a strong smoky smell or foul smell would lower prices. Similarly, several store owners told that relatively small cuts on the ventral surface were preferred to large cuts for many sea cucumber species (e.g. *T.*, *A. mauritiana*, *A. palauensis, H. scabra*). One Hong Kong store owner said that white teatfish around 15 cm in length were preferred by restaurants as a size for presenting whole on platters, and that specimens larger than ∼18 cm are subsequently lower priced. Prices will be lowered if there is salt residue on prickly redfish (*T. ananas*) or sandfish or if prickly redfish is curled. Elephant trunkfish (*H. fuscopunctata*) is low priced and apparently hard to sell because it gives a numb or itchy mouth when eaten.

**Table 2:** GLM analysis final model of effects of species, specimen length and physical damage to beche-de-mer on the (*Ln*-transformed) price per kg of beche-de-mer in Chinese stores.

Source	SS	d.f.	*F*	*p*
Species	48.46	10	27.2	<0.001
Specimen length	5.47	1	30.7	<0.001
Physical damage	0.97	2	2.7	0.072
residual	15.13	85		

## Discussion

### Export Prices and Trade from Pacific Islands

A two-fold to four-fold increase from export prices to market price for most sea cucumber species underscores the lucrativeness of trade for these commodities. The huge proportional increases in sale prices of sandfish *H. scabra* and golden sandfish *H. lessoni* compared to their export price underpin the intense demand for these two species. This also exposes why wild populations of both species are under grave threat throughout most of their geographic distribution [30]. Future research should examine the price structure of multiple species across the entire value chain.

Unlike reports that some beche-de-mer from some Pacific island countries was exported to Singapore 1–2 decades ago [15]–[17], no indication of this was presently found. The relatively minor exports to Australia are likely to regional traders who re-export to Asian markets. Hong Kong is the main market entrepôt for Pacific island beche-de-mer. This contrasts with a greater diversity of market destinations for exports from East Africa [31].

The large variation in export prices of beche-de-mer from Pacific islands is likely attributed to at least four factors: (1) the quality of post-harvest processing of the sea cucumbers; (2) potential differences in sizes of the raw sea cucumbers among countries; (3) how well connected the exporters are with the importers; (4) the nature of the supply chains.

Exports from some of the Pacific islands in this study used to be dominated by high-value and medium-value species decades ago [15] and nowadays the exporters most prefer to buy white and black teatfish (high-value) from fishers. However, most of the present export volume is low-value and medium-value species. This shift reflects a dramatic decline in stocks of valuable species through over exploitation [9], [10], most telling in the data from Fiji and Kiribati. An exception was New Caledonia, where high-value species still make up much of the export volume, indicating better sustainability of that fishery [9], [32].

### Variations in Price among Species and Product Size

This study affirms that sandfish *H. scabra* and golden sandfish *H. lessoni* are the most valuable tropical sea cucumbers in dried seafood markets in China. The price range for sandfish was greater because small sandfish are often sold and, implications for fisheries sustainability notwithstanding, the market seems to readily accept them. The price data reinforce the idea that golden sandfish should be a serious candidate for aquaculture [33] and should be cautiously managed in fisheries due to intense market pressures on exploitation [27].

Previous studies suggest that bigger beche-de-mer are worth more per unit weight [23], [25], [26] but this study shows that the relationships between price and specimen size are species specific and highly varied. Certainly some species, such as *H. scabra*, *H. lessoni*, *H. whitmaei* and *S. herrmanni* attract higher prices for larger specimens. For *H. fuscogilva* and *H. fuscopunctata*, the market appears to prefer product of medium-large size, and the small and very large products are priced lower. This trend appears to be owing to a preference by restaurants for medium-large products for presentation of whole animals on platters. For *T. ananas*, consumers apparently do not have a discernible preference for product size, so long as they are not small. Inferences from these data should be cautious since the products in Hong Kong tended to be the preferable grades and retail quantities, attracting higher prices than those in Guangzhou. Nonetheless, both markets represent Southern Chinese cuisine and the price-product length curves (Fig. 4) satisfactorily fit the data from either entrepôt.

Direct comparisons of market prices over time are difficult because there are apparently no published historical prices from Hong Kong or China. The prices in the present study are generally about 6–11 times (range: 3–19 times) the prices reported for the same species of South Pacific beche-de-mer from Singapore and Asian ports from 2001–2003 [16], [34], [35]. Over the same time period, the global average Food Price Index of seafood increased by around 60% [36]. Assuming comparable prices of beche-de-mer in Hong Kong and Guangzhou in those years, the comparisons indicate a dramatic increase in market prices of beche-de-mer over the past decade.

### Implications for Post-harvest Processing

For a given species, the most important factor governing price was the body size. The analysis also indicates a 93% chance that physical damage to products affect store prices. Damage to beche-de-mer could arise from poor post-harvest handling practices, such as leaving the sea cucumbers in the sun before cooking them, transporting them in abrasive containers or handling roughly. Using improper implements to stir the sea cucumbers or remove them from cooking pots, or smoking them too close to a fire, can also cause damage resulting in broken outer body wall of the final product [13]. Interviews with exporters revealed that fishers regularly damaged the sea cucumbers sold to them.

Although there was low representation of over-smoked and over-cut beche-de-mer, several shop owners explained that these factors would lower prices by a small amount. The placement of the cut made to gut the sea cucumbers was important for some species (e.g. *H. fuscogilva*, *H. whitmaei*, *T. ananas*) more than others (e.g. *B. argus*, *S. herrmanni*). This study therefore indicates that training in post-harvest processing should pay special attention to handling methods and processing practices that limit physical damage to the outer body wall of the sea cucumbers.

### Implications for Fisheries Management and Aquaculture

The data confirm the frequent sale of small products that represent juveniles and sub-adult sea cucumbers (c.f. [6]). The small products attracted a small fraction of the prices that the same species can sell for if the animals are harvested at a large adult size. The best example of this was for sandfish *H. scabra*, for which the non-linear relationship (Fig. 4) predicts that beche-de-mer less than 8 cm long would sell for less than US$165 kg^−1^ but beche-de-mer of 12 cm sell for US$840 kg^−1^. Hence, for species with such pricing structure, there should be an enormous advantage for strict minimum size limits in fisheries. Undersized sea cucumbers and beche-de-mer were sometimes sold to exporters in each of the four Pacific island countries, suggesting that legal size limits, better communication about size limits and/or better enforcement of the regulations are needed. Size limits for live sea cucumbers are a useful guide for fishers but enforcement should be most practical for dried beche-de-mer at processing and export hubs in producing countries [37].

The finding that prices of white teatfish and elephant trunkfish were highest at intermediate (medium-large) size suggests that both minimum and maximum size limits would be economically optimal for those species in fisheries. Such ‘slot limits’ would, in principle, prevent juveniles and young adults from being harvested and to allow very large animals to remain as breeders in the wild. However, enforcement of slot limits could be difficult in artisanal sea cucumber fisheries.

Aquaculture operations should incorporate this study’s findings into cost-benefit analyses to optimise the size at which to harvest sandfish and potentially other sea cucumber species. Production costs depend on grow-out timeframes [11], and there are environmental and sociological risks of longer grow-out periods [38]. On the other hand, the costs and risks may be offset by high sale prices if large animals can be sold and exported for premium prices.
